# The Role of Synaptopodin in Membrane Protein Diffusion in the Dendritic Spine Neck

**DOI:** 10.1371/journal.pone.0148310

**Published:** 2016-02-03

**Authors:** Lili Wang, Andréa Dumoulin, Marianne Renner, Antoine Triller, Christian G. Specht

**Affiliations:** Biologie Cellulaire de la Synapse, Inserm U1024, CNRS 8197, Institute of Biology, Ecole Normale Supérieure (ENS), Paris, France; Institute for Interdisciplinary Neuroscience, FRANCE

## Abstract

The dynamic exchange of neurotransmitter receptors at synapses relies on their lateral diffusion in the plasma membrane. At synapses located on dendritic spines this process is limited by the geometry of the spine neck that restricts the passage of membrane proteins. Biochemical compartmentalisation of the spine is believed to underlie the input-specificity of excitatory synapses and to set the scale on which functional changes can occur. Synaptopodin is located predominantly in the neck of dendritic spines, and is thus ideally placed to regulate the exchange of synaptic membrane proteins. The central aim of our study was to assess whether the presence of synaptopodin influences the mobility of membrane proteins in the spine neck and to characterise whether this was due to direct molecular interactions or to spatial constraints that are related to the structural organisation of the neck. Using single particle tracking we have identified a specific effect of synaptopodin on the diffusion of metabotropic mGluR5 receptors in the spine neck. However, super-resolution STORM/PALM imaging showed that this was not due to direct interactions between the two proteins, but that the presence of synaptopodin is associated with an altered local organisation of the F-actin cytoskeleton, that in turn could restrict the diffusion of membrane proteins with large intracellular domains through the spine neck. This study contributes new data on the way in which the spine neck compartmentalises excitatory synapses. Our data complement models that consider the impact of the spine neck as a function of its shape, by showing that the internal organisation of the neck imposes additional physical barriers to membrane protein diffusion.

## Introduction

The majority of excitatory synapses in the central nervous system are contained within dendritic protrusions called spines. The separation of the site of synaptic transmission from the dendritic shaft allows synapse-specific inputs and signal processing. This compartmentalisation relies critically on the geometry of the thin spine neck that connects the spine head with the dendrite (discussed in [1]). While some controversy remains regarding the role of spines for the electric compartmentalisation of the synapse [2,3], it has become clear that the spine neck creates a barrier to the diffusion of synaptic components (e.g. [4,5]), including membrane proteins [6,7]. The spine neck thus restricts the recruitment or the loss of key molecules involved in the induction and/or expression of synaptic plasticity such as neurotransmitter receptors [8].

Whether the biochemical coupling of the spine is mostly regulated by the geometry of the spine neck [5], or whether various sorting mechanisms control the diffusion through the neck, is not well known. A longitudinal network of branched and linear filaments of F-actin gives the spine neck its overall shape [9]. In addition, the exchange of synaptic components may be regulated by molecules or structures that are specific to the spine neck, such as septin-7 [10], the actin-binding protein synaptopodin (SP) or the spine apparatus (SA), with which it is associated (reviewed in [11]). Fluorescence recovery after photobleaching (FRAP) experiments have demonstrated that the SA in itself does not limit the diffusion of cytoplasmic components [12]. SP-deficient mice not only lack a SA but also have deficits in long-term potentiation and learning paradigms [13]. One mechanism by which SP is linked to synaptic plasticity is related to the role of the SA in calcium homeostasis and signalling [14]. Synaptic plasticity in turn can affect the morphology of the spine neck ([2,5], discussed in [4]). Here, we addressed the question whether the presence of SP in the spine neck also has a direct impact on the diffusion of membrane proteins, and if so, whether this depends on molecular interactions or on geometrical constraints.

Until recently it has been technically difficult to characterise the role of the spine neck for the biochemical compartmentalisation of the synapse, due to the limited spatial resolution of conventional fluorescence microscopy (e.g. [15]). New imaging technologies however have made it possible to combine detailed morphological analyses of spines with functional readouts and molecular dynamics (reviewed in [1]). In this study, we have used super-resolution microscopy of dendritic spines to resolve the internal organisation of the spine neck, along with single particle tracking (SPT) in order to relate the mobility of membrane proteins to the morphology of the neck. We have thus investigated the contribution of synaptopodin to the diffusion barrier regulating the exchange of membrane proteins at excitatory synapses.

## Results

### SP is present in a majority of spine necks in hippocampal neurons

In the nervous system, synaptopodin (SP) is expressed in telencephalic neurons [16] and has been shown to be specifically located in the neck of dendritic spines, where it is associated with the spine apparatus [17]. Based on this unique distribution we set out to investigate a putative role of SP as a barrier for diffusion of membrane proteins in the spine neck. To this aim, we initially characterised the sub-cellular localisation of SP in dissociated hippocampal cultures using immunocytochemistry.

**Fig 1:**
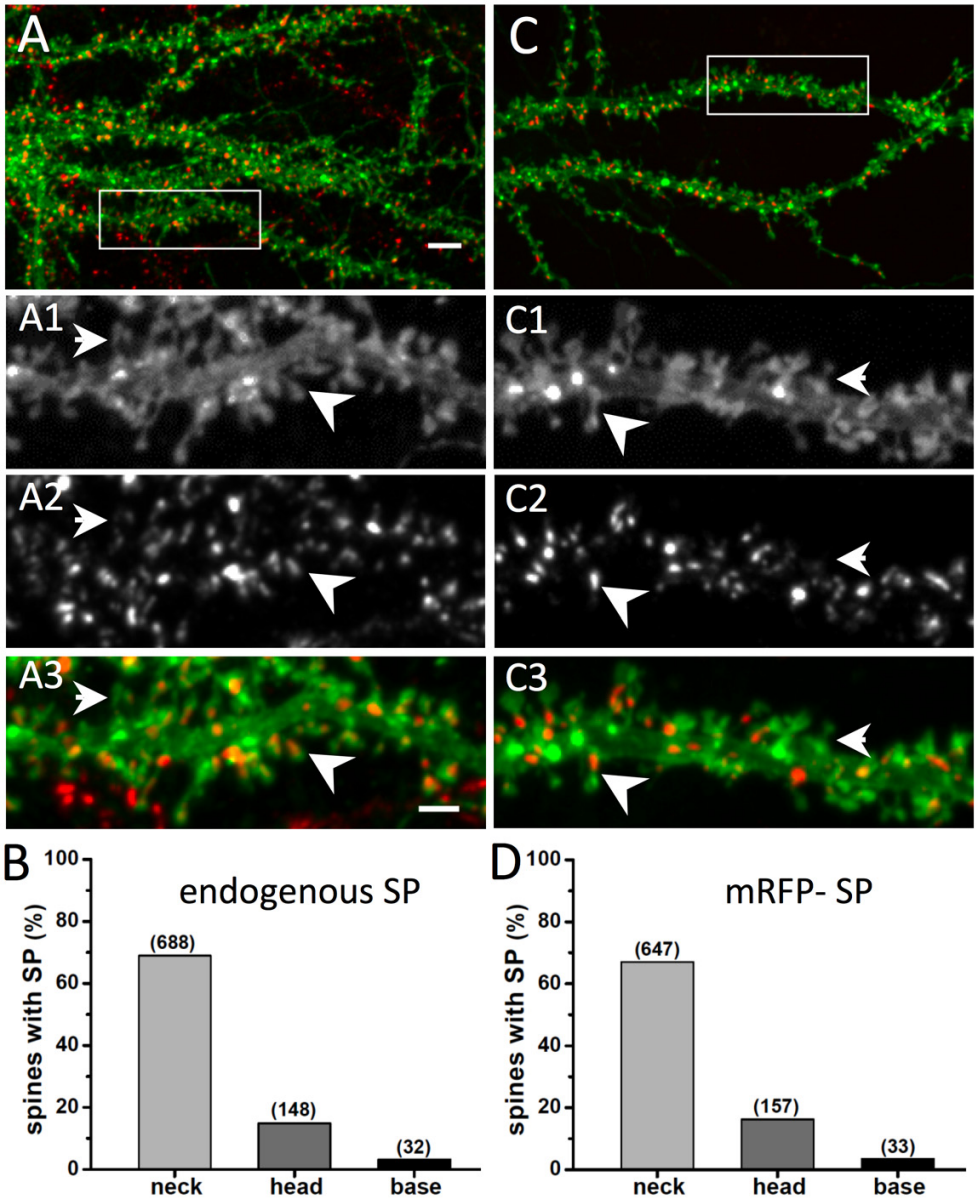
Distribution of SP in dendritic spines of hippocampal cultured neurons. (A) Localisation of endogenous SP in dissociated cultured hippocampal neurons transfected with TMD-pHluorin (green) for the visualisation of the spine morphology and immunolabelled for SP (red). Higher magnifications of the selected area (white box) are shown in A_1_ (TMD-pHluorin), A_2_ (SP) and A_3_ (merged image). The arrowhead indicates a SP cluster located in a spine neck, whereas the arrow points to a spine devoid of SP. Scale bars: 5 μm in A, 2 μm in A1-3. (B) Data quantification shows that 87.0 ± 1.1% (mean ± SD) of spines overall are positive for SP. A more refined analysis indicates a preferential distribution in the spine neck compared to head or spine base compartments (neck, 68.9 ± 1.5%; head, 14.8 ± 1.1%; base, 3.2 ± 0.6%; n = 998 spines from 3 independent experiments; Table A in S1 File). (C) Recombinant mRFP-SP distribution in hippocampal neurons. Neurons were co-transfected with TMD-pHluorin (green) and mRFP-SP (red). Zoomed images of the selected area (white box) are shown in C_1_ (TMD-pHluorin), C_2_ (SP) and C_3_ (merged image). (D) Quantitative analysis indicates that 86.7% ± 1.1% of all spines contain mRFP-SP clusters. The distribution within the neck, head and base of the spine is similar to that observed for endogenous SP (neck, 67.0 ± 1.5%; head, 16.3 ± 1.2%; base, 3.4 ± 0.6%; n = 965 spines from 4 experiments; Table A in S1 File).

For the visualisation of the cell morphology, rat hippocampal neurons were transfected with an expression construct containing a single transmembrane domain (TMD) fused at its C-terminus with an extracellular pHluorin tag. Mature hippocampal cultures at day *in vitro* (DIV) 20–23 were fixed and labelled with an antibody against SP (Fig 1A). Quantification of the SP distribution (Fig 1B) revealed that endogenous SP is present in the neck of 68.9% of the identified spines, and less frequently in the spine head (14.8%) or in the dendrite at the base of the spine (3.2%). In neurons that were co-transfected with TMD-pHluorin and an mRFP-tagged SP construct, the distribution of the recombinant protein was essentially the same, with 67% of spine necks, 16.3% of spine heads and 3.4% of spine bases containing SP (Fig 1C and 1D). These data indicate that endogenous SP and recombinant mRFP-SP display the same preferential localisation in the neck of dendritic spines. All in all, SP is expressed in a majority (close to 90%) of mature spines in cultured hippocampal neurons, which is consistent with the presence of a spine apparatus in over 80% of adult mushroom type spines [18]. These values are higher than those reported in other studies, where SP was present in approximately 30% of spines [19–21], which we attribute to the fact that our quantifications were done on clearly protruding spines with a preference for mature mushroom type spines, and in hippocampal neurons expressing detectable levels of endogenous or recombinant SP.

### SP occupies part of the inner volume of the spine neck

**Fig 2:**
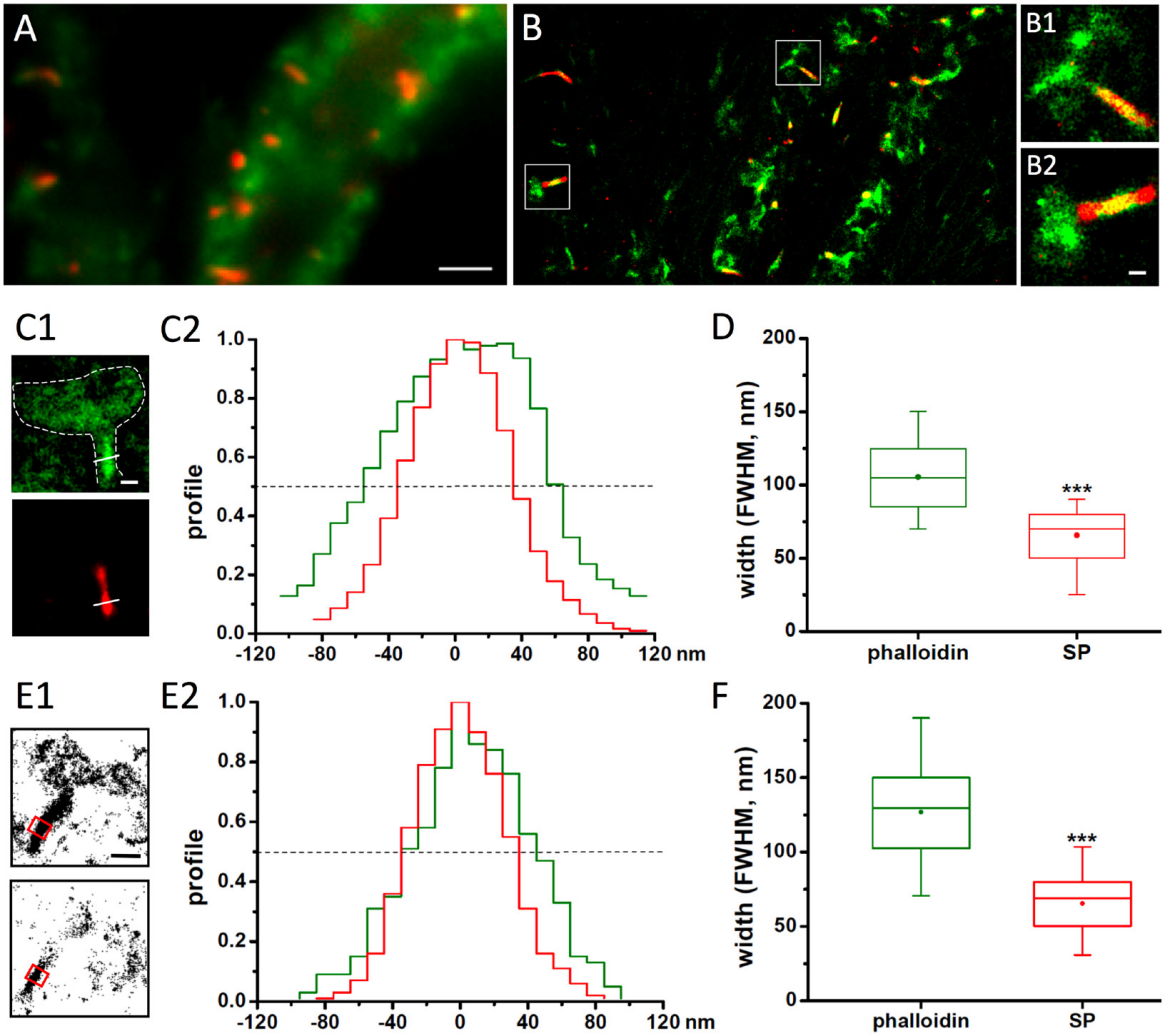
SP distribution within the spine neck. (A) Conventional fluorescence microscopy of a lentivirus-infected hippocampal neuron expressing dendra-SP (red). F-actin filaments were labelled with A647-phalloidin (green). (B) Super-resolution STORM/PALM imaging of the same dendritic segment. Single dendra and A647 fluorophore detections were rendered with a 2D Gaussian distribution with σ = 10 nm and represented in false colours (red and green, respectively). B_1_ and B_2_ are high magnifications of individual spines (white boxes in B), where SP is clearly visible along the spine neck, while phalloidin stains both neck and spine head. (C) Analysis of the full width at half maximum (FWHM) of the SP (red) and phalloidin domains (green) along a line through the spine neck in rendered STORM/PALM images. Measurements from an individual spine are shown in C1 and C2, the shape of the spine head is indicated in the upper panel in C1 (white outline based on the phalloidin staining). Note that the profile peaks in C2 were manually aligned. (D) Quantification of the FWHM of phalloidin and SP domains in spine necks. The box indicates the median, 5, 25, 75 and 95% of the spine population, the mean width is shown as a dot (n = 33 spines, 5 cultures, see also Table C in S1 File). (E,F) Analysis of SP and F-actin profiles in a 200 nm wide segment across the spine neck (red square in E1), based on the single molecule detections in pointillist images. An individual spine is shown in E1 (phalloidin: top, SP: bottom) with the corresponding detection profiles in E2. The population measurements are given in panel F. Scale bars: 2 μm in A, 200 nm in B2 and C1, 500 nm in E1.

To further characterise the sub-cellular distribution of SP, we performed super-resolution imaging of SP in dendritic spines. Hippocampal neurons were infected at DIV 1 with a lentivirus driving the expression of SP tagged with the photo-convertible fluorophore dendra2. Like recombinant mRFP-SP, the sub-cellular distribution of this construct matches that of the endogenous protein (Fig A in S1 File). Neurons were fixed at three weeks in culture and labelled with A647-phalloidin to visualise the F-actin cytoskeleton (Fig 2A). Stochastic optical reconstruction microscopy (STORM) and photoactivated localisation microscopy (PALM) of the two fluorophores were done sequentially as described in the methods section. The pointing accuracy, estimated as the standard deviation of single fluorophore detections in sparsely labelled areas, was σ_x_ = 12.2 ± 2.2 nm and σ_y_ = 12.9 ± 2.2 nm for dendra2 (mean ± SD, n = 27), and σ_x_ = 14.4 ± 4.0 nm and σ_y_ = 14.2 ± 4.4 nm for A647 (n = 21). Dual-colour super-resolution images were then generated by rendering the A647 and dendra2 detections with a Gaussian distribution with σ = 10 nm, resulting in images of SP and F-actin in dendrites with a spatial resolution of approximately 30 nm (Fig 2B).

Intensity profiles across the spine neck in rendered images revealed that the width of the SP domain was noticeably smaller than the region occupied by F-actin (Fig 2C). This suggested that the distribution of SP is limited to the inner part of the neck, whereas F-actin is present throughout the entire volume of the neck. Quantification of the full width at half maximum (FWHM) showed that the mean width ± SEM of the F-actin domain is 105 ± 4 nm (Fig 2D), in line with previous measurements of the inner spine neck diameter [22]. SP occupies a significantly smaller region of the neck with a width of 66 ± 3 nm (p < 0.001, MW test, n_1,2_ = 34 spines). The difference of 40 ± 4 nm in the width of the two domains means that there is a gap of around 20 nm between the SP domain and the inner surface of the plasma membrane. The analysis of the spine neck diameter based on single molecule detections in a 200 nm wide segment across the spine neck (Fig 2E and 2F) confirmed the narrow distribution of SP (65 ± 3, n = 36) relative to the F-actin domain (127 ± 6 nm, n = 35; p < 0.001, MW). In contrast, the outer spine neck diameter was substantially larger (195 ± 9 nm, n = 18), as judged by the detection profile of an immunolabelled membrane probe (TMD-pHluorin; Fig B in S1 File).

### SP affects the diffusion of membrane proteins in the spine neck

**Fig 3:**
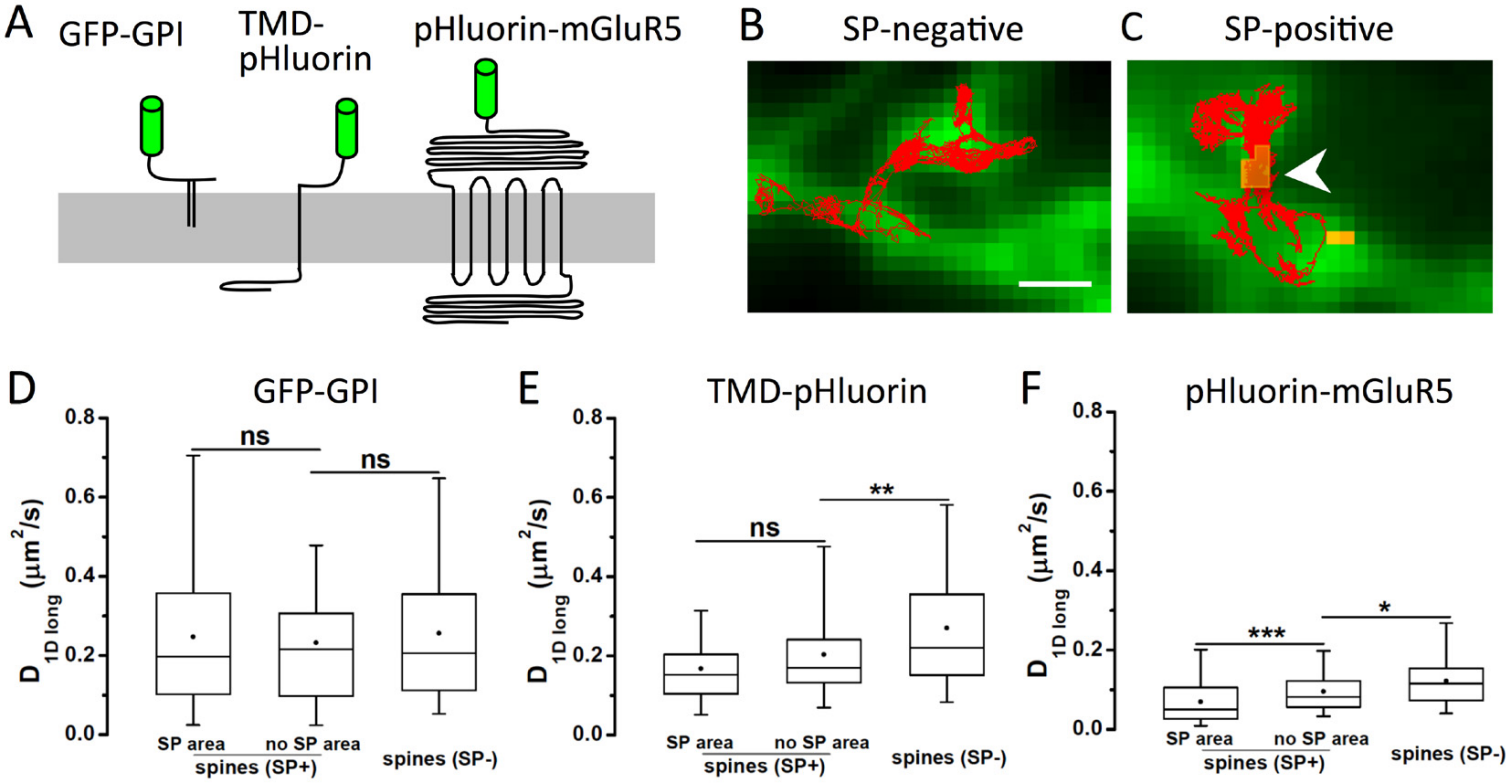
Role of SP on membrane protein diffusion in the spine neck. (A) Schematic representation of three membrane and membrane-associated constructs used in this study: GPI-anchored GFP (GFP-GPI), TMD-pHluorin with a single transmembrane domain (TMD) and a short intracellular sequence, and pHluorin-mGluR5, containing seven TMDs and a cytoplasmic domain of 352 amino acid residues (drawn to scale). All constructs have an extracellular fluorophore used for antibody coupling with quantum dots (QD). (B,C) QD trajectories were recorded in SP-negative (B) and SP-containing spines (C). Expression of pHluorin-mGluR5 is shown in green, and SP in yellow (arrowhead); scale bar: 1 μm. (D-F) Quantification of QD diffusion in spine necks for GFP-GPI (D), TMD-pHluorin (E) and pHluorin-mGluR5 (F). Trajectories were analysed either in spines negative for SP (SP-) or positive for SP (SP+). For the latter, traces on top of SP clusters (SP area) or in areas devoid of SP (no SP area) were considered separately. The diffusion coefficient was calculated on the longitudinal component of displacements along the spine neck axis (D_1Dlong_; boxes indicate 5, 25, 50, 75 and 95% of all trajectories; dots: mean value; ns: not significantly different, * p < 0.05, ** p < 0.01, *** p < 0.001, KS test; n ≥ 54 trajectories, 3–5 cultures; see also Table D in S1 File).

Given the role of the spine neck for the compartmentalisation of spiny synapses [1] we hypothesised that the presence of SP may exert an influence on the diffusion of membrane proteins through the neck. Such an effect may rely on direct or indirect interactions between the cytoplasmic domain of the membrane proteins and the molecular structures that are locally present in the neck [23]. To address these issues, we compared three membrane proteins with different membrane topologies and measured their diffusion properties in the spine neck (Fig 3A). All three constructs contain extracellular fluorophore tags to which quantum dots (QDs) were attached via specific antibodies, in order to track their diffusion in the spine head, spine neck and the neighbouring dendritic segments. The diffusion in the neck was measured in spines without SP and in spines with SP, as judged by the absence or presence of mRFP-SP that was co-expressed together with the membrane constructs (Fig 3B and 3C).

We observed that a GPI-anchored GFP protein displayed a fast and highly variable diffusion in the neck of spines, independent of the presence of SP (Fig 3D). There was no obvious difference in the diffusion coefficients of GFP-GPI in spines containing synaptopodin (SP+) and in SP-negative spines (SP-), nor in sub-regions of the neck where SP was present or not (*SP area* versus *no SP area* in SP+ spines). Note that in these experiments we measured the longitudinal component D_1Dlong_ of the diffusion coefficient in order to exclude the bias that results from the calculation of D in two-dimensional projections of a curved structure [24]. In contrast to GFP-GPI, a pHluorin-tagged membrane construct with an actual transmembrane domain and a short intracellular sequence of 36 amino acid residues (TMD-pHluorin) diffused more slowly in the spine neck (Fig 3E). Again, the speed of diffusion was not significantly different between areas with and without synaptopodin in SP+ spines, suggesting that SP does not alter the diffusion of these membrane proteins directly. However, the speed of diffusion in SP-negative spines was significantly faster than that in SP+ spines (p < 0.01, KS test, n_1_ = 68, n_2_ = 57 trajectories). This effect may be attributed to a role of SP in the internal organisation or maturation of the spine, having an indirect effect on the mobility of membrane proteins.

We also measured the diffusion of pHluorin-tagged metabotropic glutamate receptor mGluR5 in the spine neck. This receptor is enriched in dendritic spines (Fig C in S1 File, and [25]) and its structure encompasses an extracellular glutamate-binding domain followed by seven transmembrane segments and a large cytoplasmic tail of 352 residues. Given the size and the complex membrane topology of mGluR5 it is not surprising that its diffusion in the spine neck was much slower than that of the other two constructs (Fig 3F). Furthermore, we observed significant differences in D_1Dlong_ not only between SP-negative and SP-positive spines (p < 0.05, n_1_ = 89, n_2_ = 105), but also between sub-regions of spines where SP was present or absent (p < 0.001, n_1_ = 123, n_2_ = 105; see also step size analysis in Fig D in S1 File). These data suggest that synaptopodin has a specific effect on the diffusion of mGluR5 receptors, either due to a direct interaction or through an indirect mechanism that is restricted to a region close to the SP domain. An mGluR5-specific diffusion barrier has previously been demonstrated in astrocytic processes [26]. To distinguish between these possibilities we investigated the relationship between the actin cytoskeleton and SP in the spine neck as well as the downstream consequences on receptor diffusion.

### Neuronal activity increases spine neck diffusion of mGluR5 independently of SP

Hippocampal neurons were treated with 50 μM 4-aminopyridine (4AP) to increase the network activity [27]. Application of 4AP is also known to induce activity-dependent depolymerisation of F-actin [28]. We speculated that the depolymerisation of microfilaments could modulate the clustering of SP, owing to the association of SP with actin [16]. This may in turn affect the diffusion properties of mGluR5.

**Fig 4:**
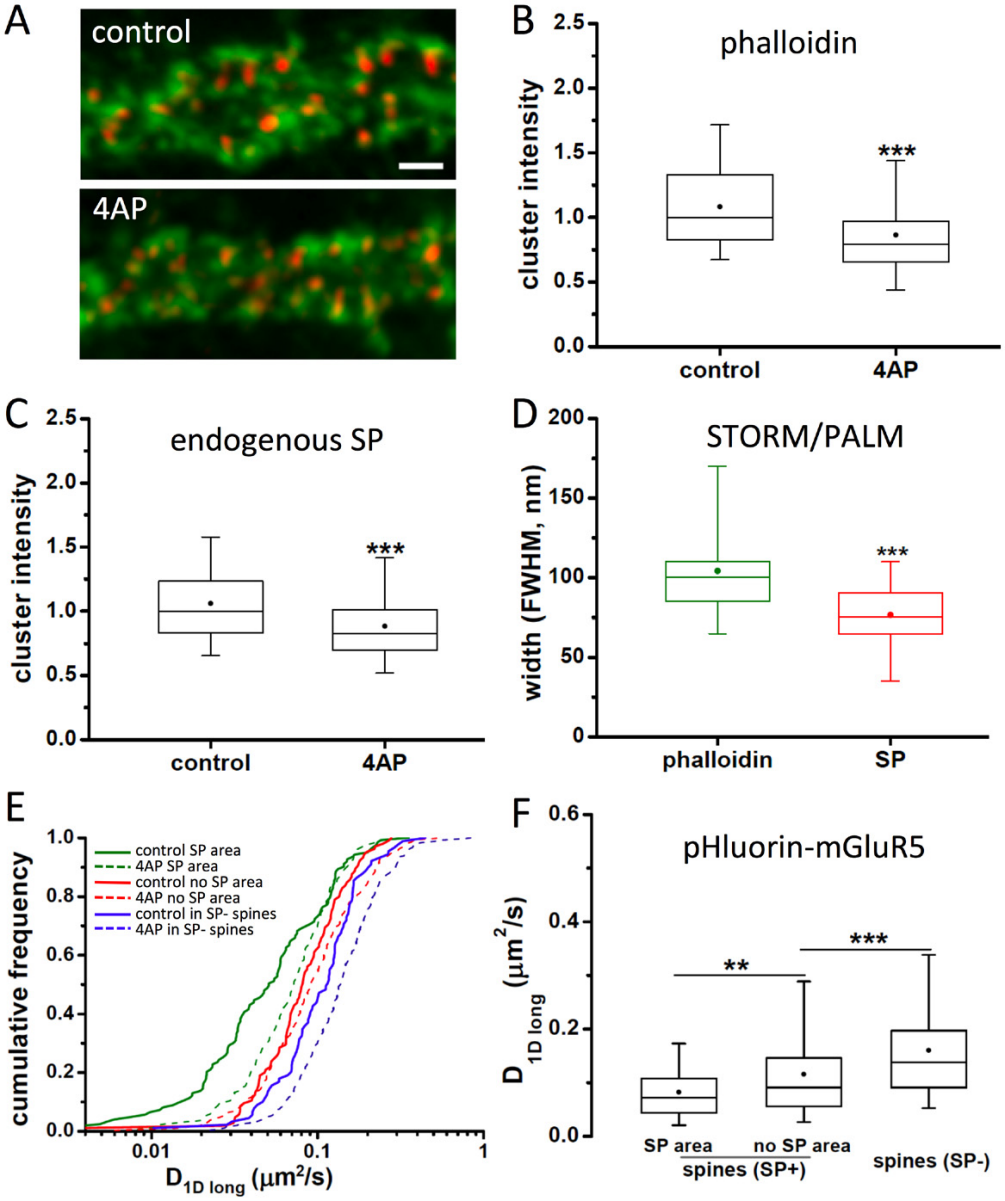
Effect of neuronal activity on SP distribution and mGluR5 diffusion. (A) Immunocytochemistry of endogenous SP (red) and A647-phalloidin staining (green) in hippocampal neurons under control conditions (top panel) or after 30 minutes of 50 μM 4AP incubation (bottom). Scale bar: 2 μm. (B,C) Normalised total fluorescence intensity of phalloidin (B) and SP levels (C) in control and 4AP treatment, quantified within SP-positive clusters (Table B in S1 File). (D) Measurements of rendered super-resolution images show no change in the widths of the SP and phalloidin domains in the spine neck after 4AP application (4AP: n = 30 spines, 7 cultures) compared to control (see Fig 2D, Table C in S1 File). (E) Cumulative distribution of pHluorin-mGluR5 diffusion coefficients tracked in SP- (blue) and in SP+ spines, either on top (green) or outside of SP clusters (red), in control (solid lines) and after 4AP treatment (dashed lines). (F) Diffusion coefficients of pHluorin-mGluR5 in SP- and SP+ spine necks after 4AP incubation (n ≥ 135 trajectories from 3–5 cultures; Table E in S1 File).

Application of 4AP for 30 minutes reduced the amount of phalloidin-labelling significantly (Fig 4A and 4B; p < 0.001, MW test, n_ctr_ = 151, n_4AP_ = 149), but had no effect on the number of SP-positive spines along the dendrites (control: 3.51 ± 0.84 spines / μm dendrite, mean ± SD; 4AP: 3.56 ± 0.74; p = 0.44, MW, n_ctr_ = 142, n_4AP_ = 144). SP intensity levels decreased in parallel with F-actin, suggesting that the structure of the spine cytoskeleton indeed has an impact on the distribution of SP (Fig 4C). Interestingly, super-resolution imaging revealed that the widths of the F-actin and the SP domains in the spine neck were not significantly altered by 4AP treatment (width of phalloidin domain, p = 0.65; SP cluster, p = 0.1, MW test, control versus 4AP, n_ctr_ = 34, n_4AP_ = 29). This shows that the internal arrangement of the spine neck is retained, even though the levels of both protein components were reduced (Fig 4D, compare with Fig 2D).

Changes in activity also had an effect on mGluR5 diffusion in the spine neck, which saw a strong increase in D_1Dlong_ values within 25–45 minutes of 4AP treatment (Fig 4E). However, this effect was recorded in all analysed neck regions, independent of the presence or absence of SP. The relative differences of diffusion in SP- spines, and in areas with or without synaptopodin in SP+ spines were preserved (Fig 4F, compare with Fig 3F), meaning that the spines retained their structural identity under these conditions. Taken together, our observations indicate that both mGluR5 diffusion and SP clustering may be regulated in response to enhanced synaptic activity and/or associated changes of the spine cytoskeleton.

### Actin depolymerisation specifically affects mGluR5 diffusion in SP-positive spines

**Fig 5:**
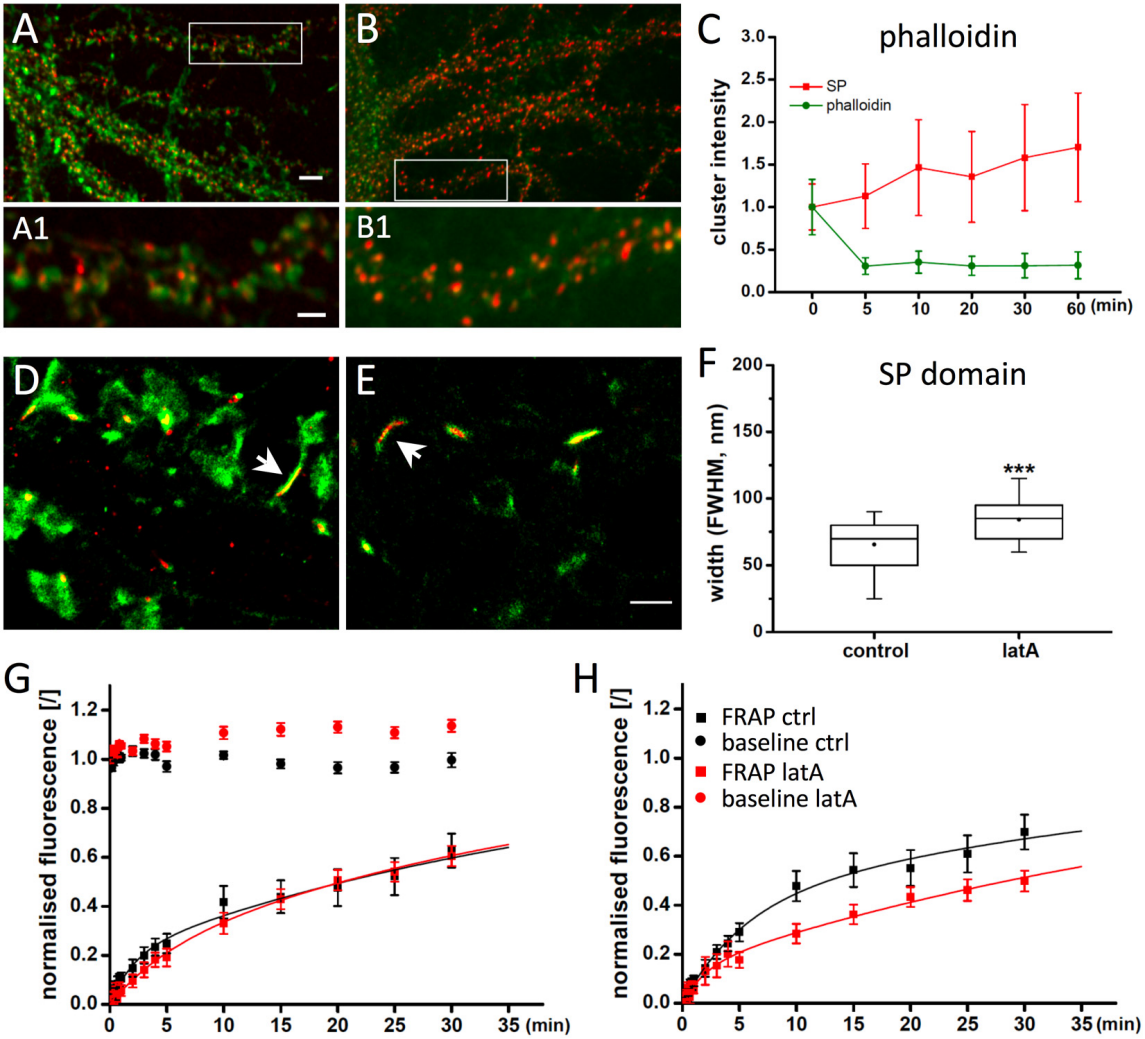
Effect of actin depolymerisation on SP distribution. (A,B) Endogenous SP (red) and A647-phalloidin (green) labelling in control neurons (A) and after 5 min incubation with 5 μM latrunculin A (B). (C) Quantification of the total phalloidin and SP fluorescence intensity in SP clusters at different time points of latA application (n ≥ 100 cells, 3 cultures, normalised mean fluorescence ± SD). All time points were significantly different from the baseline value (phalloidin: p < 0.001 for all time points versus time zero; SP: p < 0.05 at 5 min and p < 0.001 for 10–60 min versus time zero, ANOVA). (D,E) Super-resolution imaging of lentivirus-expressed dendra-SP (red) and A647-phalloidin (green) in spines in control conditions (D) and after 5 minutes latA treatment (E). (F) Quantification of the FWHM of SP domains in spine necks in control and after latA treatment (5 μM, 5 min). Actin depolymerisation induced a significant expansion of the SP domain (p < 0.001, MW, n_ctr_ = 34, n_lat_ = 33; Table C in S1 File). (G) Time-lapse imaging of mRFP-SP clusters in transfected neurons confirms the increase of cluster sizes during latA treatment (red circles) compared to control (black circles; mean ± SEM; p < 0.001 for the 15–30 min time points between the two conditions, ANOVA). FRAP recovery rates in absolute terms are not different between control (black squares) and latA (red squares; p = 0.7 for the pooled data from 10–30 min, MW). (H) Normalisation of the FRAP data to their respective baseline disclosed a reduced exchange rate during latA application (p = 0.001 for the pooled data from 10–30 min between the two conditions, MW). Scale bars: 5 μm in A, 2 μm in A1, 1 μm in D,E.

In order to induce more substantial changes in the organisation of SP clusters we challenged neurons with latrunculin A (latA) that causes rapid depolymerisation of F-actin (Fig 5A and 5B). Treatment of hippocampal neurons for 5 minutes with 5 μM latA reduced the intensity of phalloidin labelling in SP regions to approximately 35% of the initial value (Fig 5C). The drastic disruption of the F-actin cytoskeleton is not without consequence for the distribution of SP. To our surprise, total SP fluorescence increased gradually by about 70% over one hour of latA treatment. The extent of these structural changes becomes apparent in dual-colour STORM/PALM images that show near complete loss of microfilaments in the spine head and dendrites after only 5 min of latA application (Fig 5D and 5E). Phalloidin labelling is retained in the spine neck, as judged by the co-localisation with SP, and the inner diameter of the spine neck does not change, measuring 100 ± 4 nm (FWHM of the phalloidin domain, mean ± SEM; p = 0.37, MW test, n_ctr_ = 34, n_latA_ = 33). Measurements using a photo-convertible actin-binding probe (ABP-tdEosFP; [22]) confirm that the width of the actin domain in SP+ spines (112 ± 6 nm, n_ctr_ = 44) was not affected by latA treatment (106 ± 5 nm, n_latA_ = 39; p = 0.57, MW; Fig E in S1 File). The diameter of the SP domain however increased significantly (p < 0.001) from 66 ± 3 nm to 84 ± 3 nm within 5 minutes (Fig 5F). This means that the distance between SP and the inner edge of the plasma membrane is reduced to about 8 nm, instead of 20 nm in the control situation.

Time-lapse imaging revealed that SP clusters are stable over time and confirmed the recruitment of SP molecules during latA treatment (Fig 5G). The exchange rate of mRFP-SP in absolute terms was not altered by latA, as judged by fluorescence recovery after photobleaching (FRAP) experiments (Fig 5G). When the rate of recovery is expressed relative to its baseline, however, SP dynamics are significantly slowed down following latA application, illustrating an increase in SP stability in the spine neck (Fig 5H).

**Fig 6:**
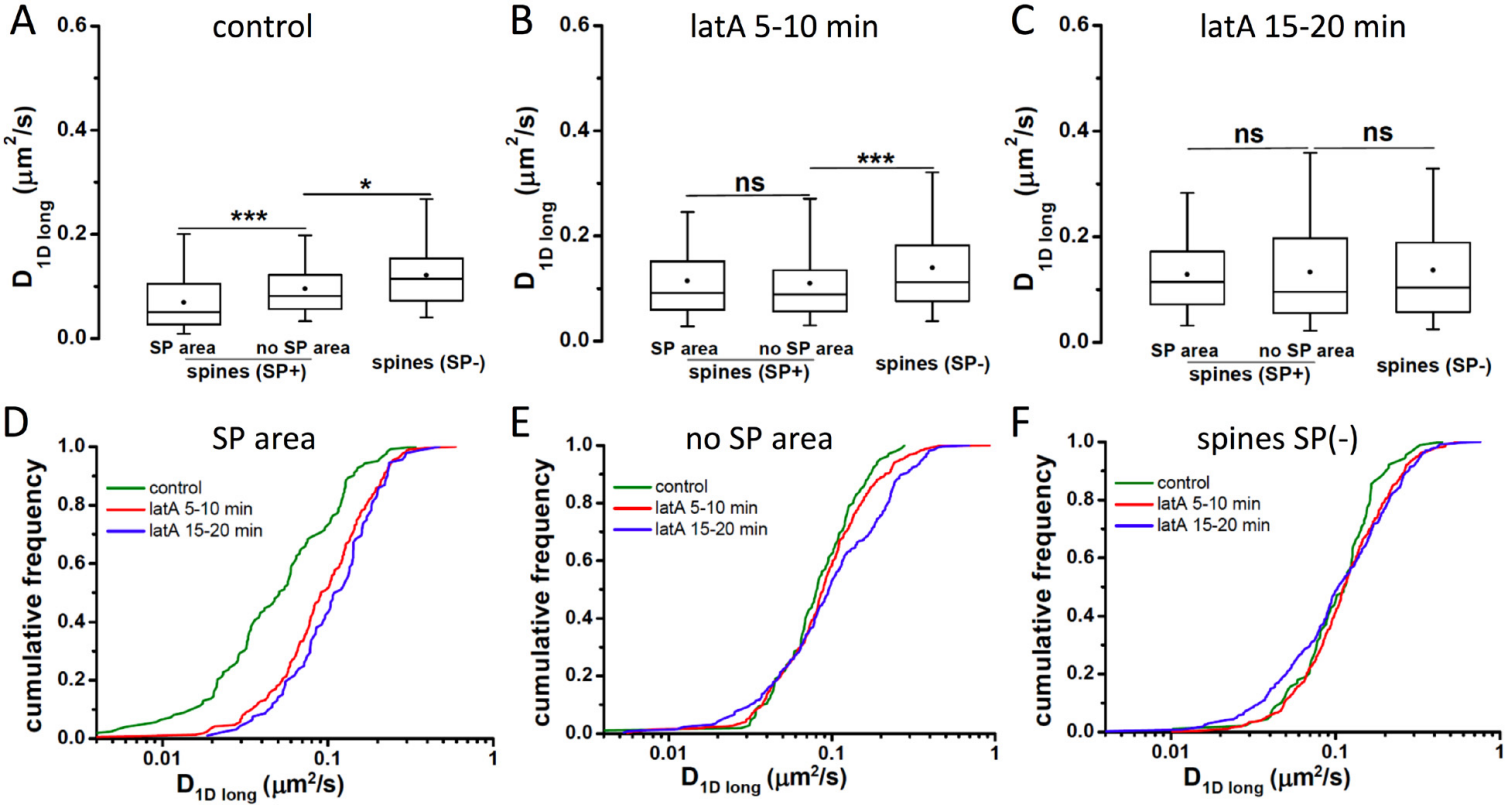
Effect of actin depolymerisation on mGluR5 diffusion. (A-C) Diffusion coefficients D_1Dlong_ of pHluorin-mGluR5 in spine necks under control conditions (A, same data as in Fig 3F), during 5–10 min (B) and 15–20 min (C) of latrunculin A exposure (n ≥ 89 trajectories from 3–5 cultures; Table E in S1 File). (D) Direct comparison of the data shown in A-C shows that the diffusion coefficients within SP domains of the spine neck increase during 5 μM latA application. (E,F) No drastic changes in diffusion occur in SP-negative regions of the spine neck (E) and in SP- spines (F) during latA treatment.

In line with the 4AP experiments, the changes in F-actin polymerisation in response to latrunculin A were paralleled by an enhanced diffusion of mGluR5 in the spine neck. However, this increase was most pronounced in SP regions of SP+ spines, to an extent that D_1Dlong_ values became indistinguishable from those in SP-free regions of the same spines within 5–10 minutes of latA application (Fig 6A and 6B). Longer latA treatment led to a further increase in mGluR5 mobility in SP+ spines, such that after 15–20 minutes the speed of diffusion was as fast as in SP- spines (Fig 6C). The temporal profile suggests that the accelerated diffusion of mGluR5 is related to the F-actin depolymerisation, rather than the result of delayed changes in the structure of the spine neck (Fig 6D, compare Fig 5C). The SP-dependence of the effect is shown by the fact that latA treatment did not alter mGluR5 diffusion in SP-free areas of SP+ spines (Fig 6E) nor in SP- spines (Fig 6F; Table E in S1 File). In conclusion, we propose that SP clustering is regulated by the actin cytoskeleton. In turn, the presence of SP correlates with a local, specific actin organisation in the spine neck of mature hippocampal neurons that acts as a diffusion barrier for neurotransmitter receptors such as mGluR5.

## Discussion

The neck of dendritic spines acts as a diffusion barrier that separates the site of synaptic transmission in the spine head from the remainder of the dendrite [1]. The thin spine neck limits the recruitment of synaptic components as well as their escape from the spine head, which has consequences for the plasticity of spiny synapses, both in terms of input-specificity and regarding the extent of any molecular changes that can occur. This is particularly relevant for the dynamic exchange of membrane proteins such as neurotransmitter receptors that are central to the induction and expression of many forms of synaptic plasticity. In this study we have therefore asked whether the diffusion of membrane proteins is shaped only by the geometry of the spine neck or whether the neck’s molecular organisation imposes additional constraints on membrane protein diffusion.

### The role of the spine neck geometry for membrane protein diffusion

Using single particle tracking (SPT) in dissociated hippocampal neurons we have observed marked differences in the diffusion behaviour of various membrane constructs in dendritic spines. The small diameter of the spine neck in itself had a limiting effect on the diffusion coefficients of membrane proteins (Fig D in S1 File, and [29]), even though the projection errors were accounted for by considering only the longitudinal component of the diffusion (D_1Dlong_). The confinement resulting from the spine neck geometry could be explained by a hydrodynamic effect due to the finite size of the membrane, as it has been described for tubular membranes with radii of 10–200 nm [29]. Alternatively, differences in membrane curvature may affect the mobility of membrane proteins differentially [30]. Yet, the diameter of the spine neck (and thus membrane curvature) in our experiments was remarkably constant as judged by the profile of the phalloidin domain in super-resolution STORM images of SP-containing spines (105 ± 25 nm, mean ± SD; Table C in S1 File), irrespective of the presence of 4-aminopyridine (4AP) or latrunculin A. This suggests that differences in neck width do not account for the diffusion properties of the various membrane proteins under these conditions. Instead, their mobility must be linked 1) to the size and complexity of the membrane proteins themselves and 2) to their interaction with other molecules in the spine neck such as F-actin and/or synaptopodin (see below).

In support of the first notion the diffusion coefficients in our SPT experiments were indeed inversely related to the size of the diffusing molecule [31]. A construct associated with the outer leaflet of the plasma membrane (GFP-GPI) displayed the fastest speed of diffusion, followed by a simple membrane protein with one transmembrane domain only (TMD-pHluorin). As expected the diffusion was slowest for the mGluR5 receptor that contains seven transmembrane domains and that furthermore forms a disulfide-bridged homodimer via its globular extracellular ligand-binding domains [32]. These findings are in agreement with previous observations showing that the exchange of AMPAR complexes and small membrane probes differed according to their size [8]. However, in the case of neurotransmitter receptors the immobilisation at binding sites within the spine head also needs to be taken into account, since it affects the diffusion properties directly (through binding), and because it reduces the apparent exchange rate by raising the local receptor population. Another factor that may influence diffusion is membrane topology. For example, proteins attached to the inner or outer leaflet of the membrane can interact differently with cellular components such as the septin-7 complex at the base of the spine neck [10]. This means that in addition to spine geometry and protein size, molecular interactions determine the kinetic properties of membrane proteins in the spine neck.

### The role of synaptopodin for membrane protein diffusion in the spine neck

Synaptopodin (SP) is preferentially located in the spine neck [17], and as such it is ideally placed to regulate the exchange of synaptic components. The presence of SP is associated with bigger spine heads [20], which in itself could increase the confinement of membrane proteins by acting as a sink with limited biochemical coupling. We have compared the diffusion properties of membrane proteins in the neck of spines expressing synaptopodin or not. In agreement with the above considerations we did not find any SP-dependent differences in the diffusion of a construct associated with the outer membrane layer. The same was true for TMD-pHluorin with its short intracellular sequence. In this context it is noteworthy that the exchange rate of cytoplasmic components was not altered in ER-positive spines, the majority of which express SP [12]. In contrast, the diffusion of the metabotropic glutamate receptor mGluR5 was slower in regions of the spine neck containing synaptopodin than in SP-free areas. A direct interaction with SP would be possible due to the long intracellular segment of mGluR5. This sequence appears to be devoid of stable globular conformations but for a number of short linear motifs (SLiMs) that are capable of adopting secondary structures upon binding to various intracellular components [33]. In other words, the flexible C-terminus would permit mGluR5 to interact with SP even though the mean distance between the plasma membrane and the SP domain was measured at 20 nm. An interaction between mGluR5 and SP could occur also via other molecules such as α-actinin [34,35]. Yet, there is evidence against such an interaction between synaptopodin and the receptor.

Firstly, mGluR5 has a sub-cellular distribution that does not fully coincide with that of SP (Fig C in S1 File). The receptor is not enriched in the spine neck, but occupies the periphery of the post-synaptic density [25]. Through its interaction with Homer, mGluR5 is linked to the synaptic scaffold of excitatory synapses [36]. This goes against the presence of mGluR5 binding sites within the spine neck. Furthermore, we have observed changes in the SP distribution and stability following latrunculin A application. Latrunculin A rapidly increased the width of the SP domain, whereby the distance to the plasma membrane was reduced from 20 nm to 8 nm. The re-distribution and recruitment of SP was accompanied by an acceleration of mGluR5 diffusion, arguing against a direct interaction between the two proteins that should have been facilitated under these conditions. A direct effect of SP on mGluR5 diffusion is therefore not likely, pointing instead to an indirect effect of SP via other structural elements within the spine neck.

### The role of the spine cytoskeleton in membrane protein diffusion

Arguably the most important element for the compartmentalisation of the spine neck is the F-actin cytoskeleton that is responsible for maintaining the shape of the spine and inducing rapid morphological changes. For example, long-term potentiation (LTP) is generally accompanied by an increase in the spine head volume, which requires the re-modelling of the actin cytoskeleton [37,38]. The organisation of F-actin in the neck is noticeably different from that of the spine head. Whereas the latter consists of a complex and highly dynamic network of branched F-actin, the spine neck has both branched as well as linear actin filaments that are arranged more or less in parallel [9]. Consequently, the neck can undergo substantial variation in length (e.g. [2,22]), while changes in width have been seen less frequently [5,39].

Given the high concentration of F-actin in the spine, it is not surprising that microfilaments below the plasma membrane obstruct the movement of membrane proteins [23]. This is consistent with our observation that application of 4AP, which causes activity-dependent depolymerisation of F-actin [28] accelerated mGluR5 diffusion in all spines, independent of the presence of synaptopodin. Saying that, the insignificant increase of mGluR5 diffusion in the neck of SP-negative spines after exposure to latrunculin A suggests that the polymerisation state of actin as such has a minor role in the regulation of spine neck diffusion. Instead, activity-dependent signalling via second messengers, kinases or phosphatases could be in part responsible for the increased mGluR5 mobility in response to 4AP, possibly by modulating interactions of mGluR5 and its binding partners [33]. In contrast, the observation that latrunculin A treatment caused a rapid and pronounced acceleration of mGluR5 diffusion in SP-containing regions of the spine neck points to a specific organisation of the F-actin cytoskeleton in these areas.

### Synaptopodin as an organiser of the spine neck

SP has been implicated in functional and structural mechanisms of synaptic plasticity [11]. The fact that SP is an essential component of the spine apparatus [13] suggests that its primary function is to stabilise this organelle in mature spine necks, possibly by mediating its attachment to the F-actin cytoskeleton. The spine apparatus is involved in calcium homeostasis and signalling (reviewed in [14]). Likewise, concurrent changes in SP clustering and the stack size of the spine apparatus were observed during homeostatic plasticity [40].

Our findings lend support to a model whereby synaptopodin acts as an organiser of the spine neck in mature spines. The spine necks are significantly wider when there is SP (Fig E in S1 File). Its presence correlates with the reduction of mGluR5 diffusion in the spine neck as a consequence of the local organisation of the spine cytoskeleton, which is reflected in the specific effect of latrunculin A in SP-positive regions of the spine neck. Although SP interacts with actin and the actin-associated protein α-actinin [16,34], our data show that SP occupies a different sub-domain of the spine neck, in line with its close association with the spine apparatus [17]. In other words, SP cannot interact with the totality of actin filaments in the neck. Rather, SP appears to associate with a pool of F-actin that is activity-dependent, as seen in the correlated reduction of F-actin and SP levels in the presence of 4AP. In contrast, the latrunculin A sensitive dynamic pool of F-actin [39] had an inverse effect on the SP domain, obstructing the accumulation of SP within the spine neck. These observations can be reconciled if we assume that the presence of SP in the spine neck creates a dense local F-actin network that blocks the recruitment of further SP molecules. This may be the result of the increased crowding in those spine necks that contain synaptopodin and a spine apparatus. Synaptopodin would thus exert an indirect effect via F-actin on the diffusion of membrane proteins in the spine neck.

## Methods

### Expression constructs

The GFP-GPI plasmid was kindly provided by Dr. S. Mayor (India, Bangalore; Sharma et al. Cell, 2004). The TMD-pHluorin plasmid driving the expression of a single transmembrane domain (TMD) from syntaxin fused to an extracellular (C-terminal) pHluorin tag was described in Ribrault et al. [41]. The pHluorin-mGluR5 plasmid containing the coding sequence of rat mGluR5a (isoform 1, UniProt accession number P31424-2) with extracellular (N-terminal) myc and pHluorin tags was generated from plasmid pcDNA3.1-Myc-mGluR5a-Venus [42]. The mRFP-SP construct contained the mouse synaptopodin (SP) sequence (neuronal isoform 3, UniProt Q8CC35-3) and was derived from pEGFP-SYNPO [34] by replacing the N-terminal tag by mRFP. Similarly, the dendra2 sequence was introduced to obtain plasmid dendra-SP. For viral infection the dendra-SP coding sequence was transferred into the FUGW replicon plasmid to generate FU-dendra-SP, used to produce lentiviral particles as described previously [43].

### Cell culture and transfection

All animal procedures were carried out according to the European Community Council directive of 24 November 1986 (86/609/EEC), the guidelines of the French Ministry of Agriculture and the Direction Départementale des Services Vétérinaires de Paris (Ecole Normale Supérieure, Animalerie des Rongeurs, license B 75-05-20), and were approved by the Comité d’Ethique pour l’Expérimentation Animale Charles Darwin (licence Ce5/2012/018). All efforts were made to minimise animal suffering and to reduce the number of animals used. Sprague Dawley rat embryos (embryonic day 18–20) were removed following CO_2_ euthanasia, decapitated, and the hippocampi dissected. Primary cultures of dissociated neurons were prepared as described [24]. Neurons were plated at a density of 2.5 x 10^4^ cells/cm^2^ onto 18 mm diameter glass coverslips pre-coated with 80 μg/ml poly-D,L-ornithine (Sigma), and maintained at 36°C in a 5% CO_2_ humidified incubator in Neurobasal medium supplemented with 2% B27 supplement, 2 mM L-glutamine, and antibiotics (all from Invitrogen).

For transfection, neurons at days *in vitro* (DIV) 9–12 were incubated for 30 min in Neurobasal medium containing 2 μL Lipofectamine 2000 (Invitrogen) and 0.5 μg plasmid DNA in a volume of 600 μL. For double transfections, 0.25 μg of each construct was used. For super-resolution experiments, neurons were infected at DIV 1 with lentivirions driving the expression of dendra-SP. After transfection/infection procedures, neurons were maintained in the incubator until used for experiments at DIV 20–23.

### Drug treatments

Latrunculin A (latA) and 4-aminopyridine (4AP) were obtained from Tocris Bioscience and used at a concentration of 5 μM and 50 μM, respectively. For immunocytochemistry and super-resolution STORM/PALM imaging, drugs were added directly to the culture medium (5–60 min) prior to fixation. In the SPT experiments, cells were pre-incubated for 20 min with 4AP in culture medium, labelled with antibody-coupled quantum dots (QDs) for 5 min (see below) and then imaged in MEM recording medium (see below) for up to 20 min in the presence of 4AP. LatA was added to the medium at the beginning of the SPT recording session that lasted for up to 20 min.

### Immunocytochemistry

Cells were fixed at room temperature for 10 min in paraformaldehyde (PFA, 4% w/v) in PBS, and permeabilised for 4 min with Triton X-100 (0.25% v/v). Cells were then incubated for 30 min in blocking solution (PBS containing 0.25% w/v gelatin, Sigma) and for 1 hour with guinea pig anti-synaptopodin antibody (1:500, 163004, Synaptic Systems) diluted in 0.125% gelatin-PBS. Alexa Fluor 488 (A488)-conjugated donkey anti-guinea pig antibody (1:1000, Jackson ImmunoResearch Laboratories) was used as secondary antibody. Incubation for 45 min with Alexa Fluor 647 (A647)-phalloidin (0.2 μM, A22287, Invitrogen) was used to label actin filaments.

Acquisition of fluorescence images was done on a spinning disk confocal microscope (LeicaDM5000B, Leica Microsystems, with a Yokogawa CSU10 spinning disk head), equipped with a CCD camera (Coolsnap, Princeton Instruments) and controlled by Metamorph software (Molecular Devices). Images were taken with a Photometrics 63x immersion objective, and exposure times were kept constant and were such as to capture the full intensity range in each channel. The quantification of the sub-cellular distribution of SP (data in Fig 1) was done manually on spines that could be clearly identified in two-dimensional projections and that were parallel to the focal plane. The counting was exclusive, meaning that SP clusters were judged to be located either in the spine head, the neck or on the dendrite at the base of the spine. For intensity measurements (data shown in Figs 4A–4C and 5A–5C), images were filtered by wavelet segmentation using an interface implemented in Metamorph [44] to generate binary masks of SP. The integrated intensity of SP and phalloidin was then measured in SP mask regions using homemade software (ImAnalysis [45]) in Matlab (MathWorks). Quantification of SP cluster densities was done on portions of dendrites (length > 10 μm) with clearly protruding spines.

### Time-lapse imaging and fluorescence recovery after photobleaching (FRAP)

Individual clusters of mRFP-SP in transfected neurons were bleached with a 561 nm laser pulse and their recovery recorded over time. Time-lapse imaging of non-bleached clusters served to monitor the degree of bleaching due to image acquisition and to quantify systematic changes of baseline levels during latA treatment. Background-corrected total intensity values (expressed as mean ± SEM) were normalised to the baseline level at time zero before bleaching (set to 1), the first time point after photobleaching (set to zero) and, where indicated (in Fig 5H), also to the respective baseline data. FRAP data were fitted with an exponential decay function with two time constants as described previously [43].

### Single particle tracking using quantum dots

To track membrane protein diffusion, quantum dots (QDs) were attached to their extracellular fluorophore tags, as reported previously [46]. Briefly, 50 nM goat anti-rabbit F(ab’)_2_-tagged QDs emitting at 655 nm (Q11422MP, Invitrogen) were incubated first with polyclonal rabbit anti-GFP antibody (1:10; 132002, Synaptic Systems) for 30 min in PBS, and then blocked for 15 min with casein in a final volume of 10 μl. Transfected neurons were incubated with the pre-coupled QDs (1:6000–1:10000 final QD dilution) for 5 min at 37°C in MEM recording medium (phenol red-free minimal essential medium, supplemented with 33 mM glucose (Sigma), 20 mM HEPES, 2 mM glutamine, 1 mM sodium pyruvate, and 2% B27, all from Invitrogen) and rinsed.

Neurons were imaged for up to 30 min in MEM recording medium in an open chamber mounted onto an inverted microscope (IX71, Olympus) equipped with an oil-immersion objective (Olympus, 63x, NA 1.45). Fluorescence was detected using a xenon lamp, appropriate emission filters (GFP: excitation 485/20, emission 535/30; RFP: ex 560/25, em 605/15; QD: ex 460/60, em 655/15; Semrock) and a charge-coupled device (CCD) camera (Cascade 512B, Roper Scientific). QD trajectories were recorded with an exposure time of 12 ms over 5000 image frames (1 min streamed recording) with Metamorph software (Molecular Devices).

QDs trajectories in synaptopodin-negative (SP-) or SP-expressing spines (SP+) were identified based on the presence of mRFP-SP clusters. To distinguish between SP-free and SP-containing areas of SP+ spines, trajectories were classified according to their co-localisation with binary mRFP-SP images (wavelet segmentation, [44]). Tracking and analysis were done as described [47] using the homemade software SPTrack_v4 in Matlab (MathWorks). The centre of the QD signal was determined with a spatial accuracy of 10–20 nm by Gaussian fitting. The spots in a given frame were associated with the maximum likely trajectories estimated on previous frames of the image sequence. Only trajectories with at least 15 consecutive frames were considered for quantification. The mean square displacement (MSD) was calculated using MSD(*ndt*) = (N−n)^-1^∑^N-n^_i = 1_[(x_i+n_−x_i_)^2^+ (y_i+n_−y_i_)^2^] *dt*, where x_i_ and y_i_ are the coordinates in frame i, N is the total number of steps in the trajectory, dt is the time between two successive frames and *ndt* is the time interval over which displacement is averaged [48]. Since membrane curvature can affect the diffusion measurement, we calculated one-dimensional MSDs taking into account only the displacement in the direction of the cylinder axis to avoid the projection errors [24]. D_1Dlong_ was calculated by fitting the points 2 to 5 of the MSD plot versus time interval with the equation MSD(t) = 2Dt + b. Given the localisation accuracy, trajectories with D_1Dlong_ < 10^−4^ μm^2^/s were considered immobile.

### Super-resolution STORM/PALM imaging

We used a combination of stochastic optical reconstruction microscopy (STORM) and photoactivated localisation microscopy (PALM), to study the distribution of A647-phalloidin-labelled actin filaments and of dendra-SP respectively. Dual-colour single-molecule imaging was carried out sequentially, as described previously [22] on an inverted Nikon Eclipse Ti microscope with a 100x oil-immersion objective (NA 1.49), an additional 1.5x lens, and an Andor iXon EMCCD camera (image pixel size, 107 nm). First, low-resolution conventional fluorescence images of the non-converted form of dendra-SP and of A647-phalloidin were taken with a mercury lamp and specific filters (dendra: ex 560/25, em 607/36; A647: ex 650/13, em 684/24; Semrock). Then, we reversibly switched A647 fluorophores between the dark and the fluorescent state in reducing buffer conditions (10% glucose in PBS containing with 50 mM ß-mercaptoethylamine (cysteamine), 0.5 mg/ml glucose oxidase and 40 μg/ml catalase from Sigma, and degassed with N_2_; [49]) under continuous 532 nm and 633 nm laser illumination (em 684/24). Subsequently, dendra fluorophores were converted and imaged by PALM, using 405 nm and 561 nm lasers (em 607/36). Generally movies of 20000 frames were acquired at frame rates of 50 ms (A647) and 100 ms (dendra). The z position was maintained during acquisition by a Nikon perfect focus system.

Super-resolution image reconstruction was done by fitting the point spread function of spatially separated fluorophores with a 2D Gaussian distribution [22]. The x/y drift during image acquisition was corrected in both channels using 100 nm TetraSpeck beads (T-7279, Invitrogen). STORM and PALM images were rendered by superimposing the coordinates of single-molecule detections, which were represented by 2D Gaussian curves of unitary intensity and with a width representing the localisation accuracy (σ = 10 nm). The full width at half maximum (FWHM) of SP clusters and F-actin profiles across the spine neck was measured in dual-colour images that were aligned manually based on the positions of the fiducial markers. Alternatively, the FWHM of the SP and F-actin domains were measured using the distribution of single molecule detections within a 200 nm wide segment across the spine neck (in Fig 2E and 2F).

### Statistical analysis

All experiments were performed in three or more independent cultures. Data obtained by immunocytochemistry and STORM/PALM were compared using the Mann-Whitney U-test (MW) or non-parametric Kruskal-Wallis one-way ANOVA together with Dunn’s multiple comparison test. Diffusion data were analysed using the Kolmogorov–Smirnov test (KS). Data values and statistical analyses are summarised in Tables A-E in S1 File. Data are generally given as mean ± standard deviation (SD) or standard error of the mean (SEM), or as median, 25% and 75% of the population.

## Supporting Information

S1 File **Figure A.** Distribution of lentivirus expressed dendra-SP. **Figure B.** SP distribution within the spine neck. **Figure C.** Overlapping localisation of mGluR5 and SP in dendritic spines. **Figure D.** Role of neck width and SP for membrane protein diffusion in the spine neck. **Figure E.** Organisation of the actin cytoskeleton in SP+ and SP- spines. **Table A.** Fluorescence microscopy data. **Table B.** Fluorescence microscopy data. **Table C.** STORM/PALM data. **Table D/E.** SPT data.
(PDF)
