# Supplementary material for: The Role of Synaptopodin in Membrane Protein Diffusion in the Dendritic Spine Neck
Source: PLoS One. 2016 Feb 3;11(2):e0148310. doi: 10.1371/journal.pone.0148310 (PMC4739495; doi:10.1371/journal.pone.0148310)
Supplement: S1 File — Figure A. Distribution of lentivirus expressed dendra-SP. Figure B. SP distribution within the spine neck. Figure C. Overlapping localisation of mGluR5 and SP in dendritic spines. Figure D. Role of neck width and SP for membrane protein diffusion in the spine neck. Figure E. Organisation of the actin cytoskeleton in SP+ and SP- spines. Table A. Fluorescence microscopy data. Table B. Fluorescence microscopy data. Table C. STORM/PALM data. Table D/E. SPT data. (PDF) [file pone.0148310.s001.pdf]

S1 Supporting information

The Role of Synaptopodin in Membrane Protein Diffusion in the Dendritic Spine Neck

Lili Wang[1], Andréa Dumoulin[1], Marianne Renner[1], Antoine Triller[1]*, Christian G Specht[1]

[1] Biologie Cellulaire de la Synapse, Inserm U1024, CNRS 8197, Institute of Biology, Ecole Normale Supérieure (ENS), Paris, France

* Corresponding author: E-mail: triller@biologie.ens.fr (AT)

Contents:

Figures A-E

Tables A-E

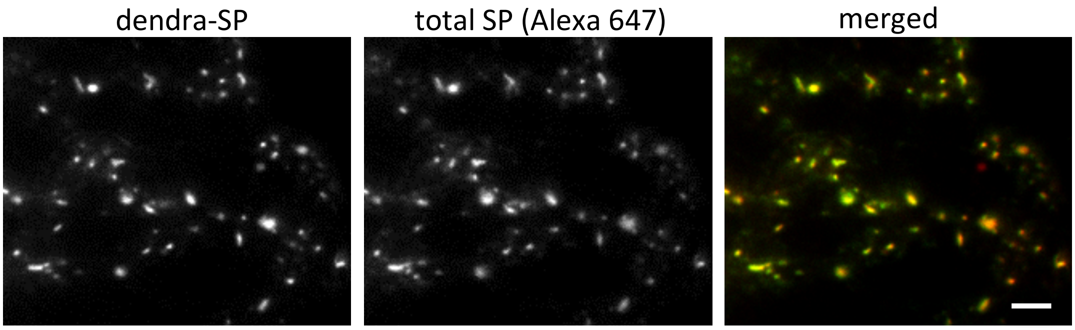

Figure A. Distribution of lentivirus expressed dendra-SP. Hippocampal neurons were infected at DIV 7 and fixed at DIV 20, followed by immunolabelling of SP using Alexa Fluor 647-conjugated secondary antibody. The distribution of recombinant dendra-SP (green) matches the total SP staining (= dendra-SP + endogenous SP, red), indicating that both proteins occupy the same sub-cellular compartments. Scale bar: 500 nm.

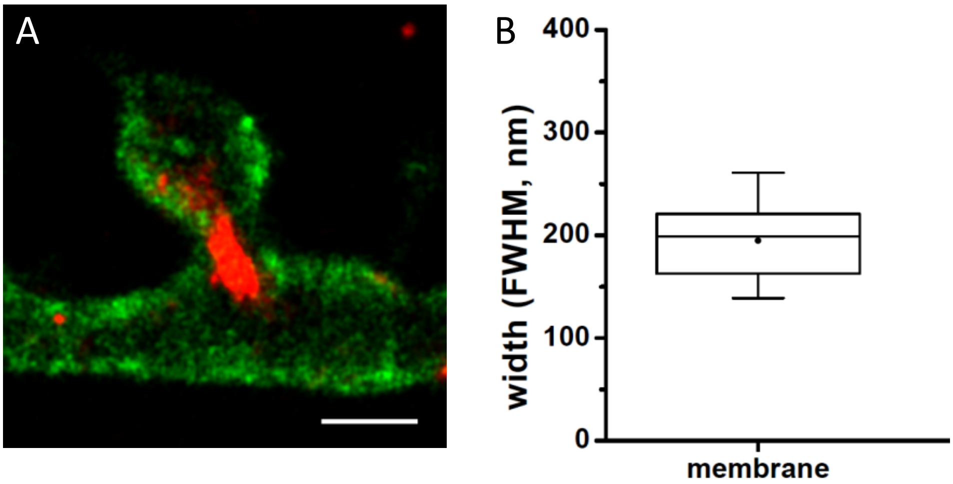

Figure B. SP distribution within the spine neck. (A) Hippocampal neurons that had been co-transfected with dendra-SP and TMD-pHluorin plasmids at DIV 9 were labelled with primary antibodies against GFP tagged with Alexa-Fluor 647 fluorophores and imaged by dual-colour STORM/PALM. (B) Quantification of the outer spine neck diameter was based on the distribution of single molecule detections of the membrane probe in a 200 nm wide segment across the spine neck (measured as the full width at half maximum, FWHM, n = 18 spines from two neurons). Scale bar: 500 nm.

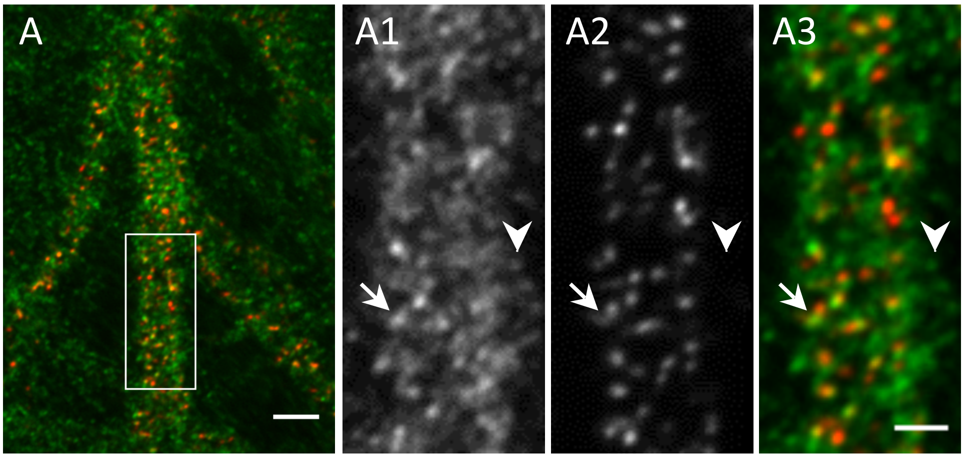

Figure C. Overlapping localisation of mGluR5 and SP in dendritic spines. Hippocampal neurons were immunolabelled with specific antibodies against SP (shown in red) and mGluR5 (green; monoclonal mouse anti rat mGluR5b, 1:1000, clone N75/33, NeuroMab, UC Davis/NIH). Endogenous mGluR5 is distributed widely, with a preference for the spine head of SP-positive spines (arrows) and somewhat less for SP-negative spines (arrowheads). We observed no specific accumulation of mGluR5 in the spine neck, arguing against a direct interaction with endogenous SP. Scale bars: 5 µm in A, 2 µm in A1-3.

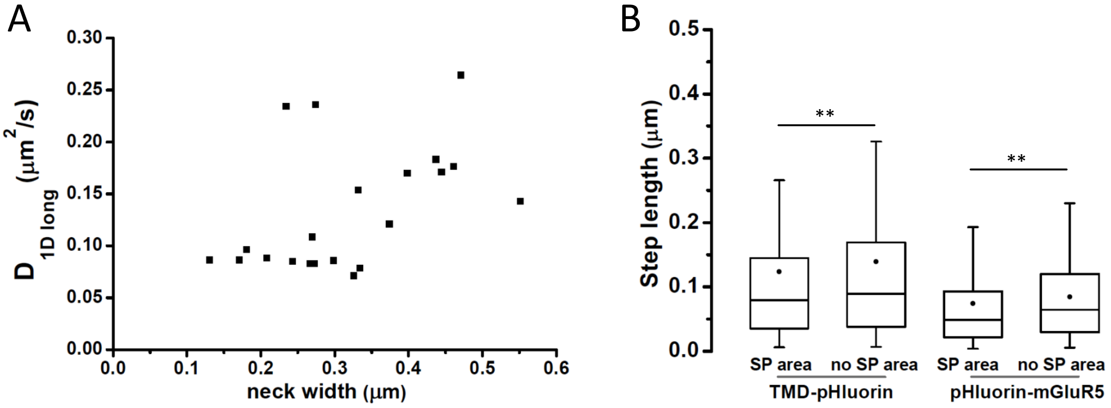

Figure D. Role of neck width and SP for membrane protein diffusion in the spine neck. (A) Correlation between longitudinal diffusion coefficients of pHluorin-mGluR5 and the spine neck diameter (measured as the width of QD detections in the neck region). (B) Step size analysis of TMD-pHluorin and pHluorin-mGluR5 in regions containing SP or without SP within the same spine necks. The longitudinal step length was calculated for 60 ms intervals (5 frames of 12 ms) and was found to be significantly smaller in SP areas of the spine neck compared to no SP areas (TMD-pHluorin: SP area, 124 ± 11 nm mean ± SEM; no SP, 139 ± 12 nm; pHluorin-mGluR5: SP area, 74 ± 4 nm; no SP, 85 ± 1 nm; n > 3800, ** $p < 0.01$, MW).

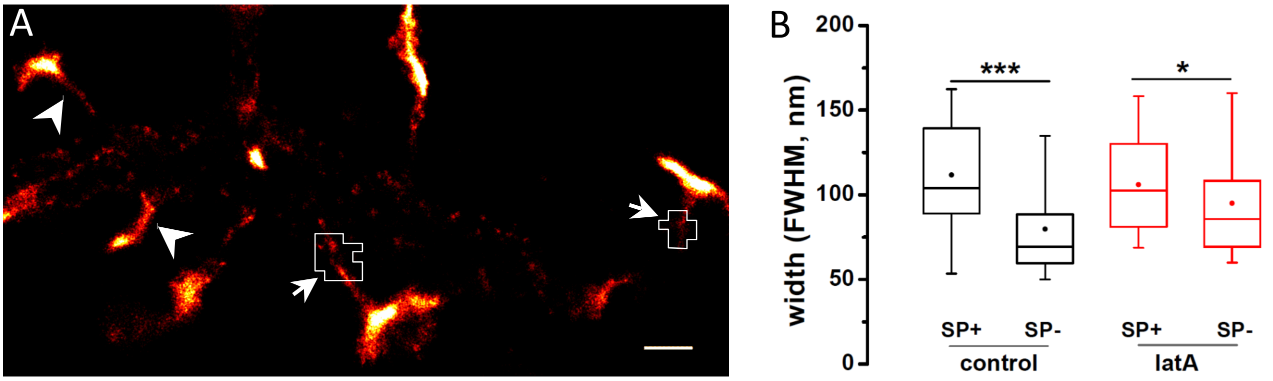

Figure E. Organisation of the actin cytoskeleton in SP+ and SP- spines. (A) PALM experiments were done in hippocampal neurons expressing the photo-convertible actin probe ABP-tdEosFP (Izeddin et al. 2011, PLoS One). SP- (arrowhead) and SP+ spines (arrows) were distinguished on the basis of co-transfected mRFP-SP by conventional fluorescence microscopy (white boxes). Note that the red mRFP fluorescence was bleached prior to PALM imaging of ABP-tdEosFP. Scale bar: 500 nm. (B) Quantification of the width of the actin domain (FWHM of single molecule detections in 200 nm wide segments across the spine neck) in SP-positive and SP-negative spines of the same neurons, under control conditions and after 5 min latA treatment. The width of SP- spines was consitently smaller than that of SP+ spines (control: SP+, 112 ± 6 nm, mean ± SEM, n = 44; SP-, 80 ± 6 nm, n = 29; latA: SP+, 106 ± 5 nm, n = 39; SP-, 95 ± 10 nm, n = 24; * $p < 0.05$, *** $p < 0.001$, MW).

Table A. Fluorescence microscopy data. Quantification of SP distribution in dendritic spines in hippocampal neurons.

Condition	n (spines counted)	Spines with SP				
		location	n	SD	percent (%)	SD (%)
**Endo-SP**	998	neck	688	14.6	68.9	1.5
		head	148	11.2	14.8	1.1
		base	32	5.6	3.2	0.6
		**total**	**868**	**10.6**	**87.0**	**1.1**
**mRFP-SP**	965	neck	647	14.6	67.0	1.5
		head	157	11.5	16.3	1.2
		base	33	5.6	3.4	0.6
		**total**	**837**	**10.5**	**86.7**	**1.1**

Table B. Fluorescence microscopy data. Effect of 4AP on SP clusters.

Condition		n (clusters)	Mean	SEM	Median	25% quartile	75% quartile	Mann Whitney U Test (control versus 4AP)
**SP density**	control	142	3.512	0.070	3.380	2.922	4.020	p=0.442
	4AP	144	3.558	0.062	3.451	3.008	4.080	
**normalized SP intensity**	control	149	1.060	0.025	1	0.832	1.232	p<0.001
	4AP	151	0.886	0.024	0.824	0.696	1.010	
**normalized phalloidin intensity**	control	149	1.083	0.028	1	0.825	1.331	p<0.001
	4AP	151	0.864	0.031	0.793	0.658	0.970	

Table C. STORM/PALM data. Distribution of SP and F-actin in the spine neck (full width at half maximum, FWHM; nm).

FWHM / Condition	N (spines)	Domain	Mean	SEM	Median	25% quartile	75% quartile	Mann Whitney U Test: phalloidin vs SP	Mann Whitney U Test: control vs treatment	
									phalloidin	SP
control	34	phalloidin	105.44	4.28	105	85	125	p<0.001		
		SP	65.58	3.41	70	50	80			
4AP	29	phalloidin	104.13	5.31	100	85	110	p<0.001	p=0.652	p=0.101
		SP	76.37	4.66	75	65	90			
Latrunculin A	33	phalloidin	100.00	4.01	100	85	115	p<0.01	p=0.373	p<0.001
		SP	84.09	3.11	85	70	95			

Table D. SPT data. Diffusion coefficient D_{1Dlong} ($\mu m^2/s$) of membrane constructs in spine neck.

D_{1Dlong} / Molecules	Area in spines	n (trajectories)	Mean	SEM	Median	25% quartile	75% quartile	Kolmogorov-Smirnov test: spine SP (+) in vs no SP area	Kolmogorov-Smirnov test: no SP area SP (+) vs SP (-)
GFP-GPI	in SP area SP (+)	164	0.247	0.0131	0.197	0.102	0.357	p=0.063	p=0.118
	no SP area SP (+)	170	0.232	0.0107	0.216	0.097	0.306		
	SP (-)	268	0.256	0.0091	0.206	0.112	0.355		
TMD-pHluorin	in SP area SP (+)	58	0.168	0.0119	0.153	0.104	0.203	p=0.551	p<0.01
	no SP area SP (+)	57	0.203	0.0180	0.17	0.132	0.241		
	SP (-)	68	0.270	0.0210	0.22	0.152	0.356		
pHluorin-mGluR5	in SP area SP (+)	123	0.070	0.0054	0.051	0.027	0.105	p<0.001	p<0.05
	no SP area SP (+)	105	0.096	0.0054	0.082	0.057	0.122		
	SP(-)	89	0.122	0.0077	0.115	0.073	0.154		

Table E. SPT data. Diffusion coefficient D_{1Dlong} ($\mu m^2/s$) of pHluorin-mGluR5 in control condition and pharmacological treatments.

D_{1Dlong} / Condition	Area in spines	n (trajectories)	Mean	SEM	Median	25% quartile	75% quartile	Kolmogorov-Smirnov test				
								spine SP (+)	no SP area	control vs treatment		
								in vs no SP area	SP (+) vs SP (-)	SP (+) spine		spine SP (-)
										in SP	no SP	
control	in SP area SP (+)	123	0.070	0.0054	0.051	0.027	0.105	p<0.001	p<0.05			
	no SP area SP (+)	105	0.096	0.0054	0.082	0.057	0.122					
	SP (-)	89	0.122	0.0077	0.115	0.073	0.154					
4AP	in SP area SP (+)	135	0.082	0.0045	0.072	0.044	0.108	p<0.01	p<0.001	p<0.001	p=0.275	p<0.01
	no SP area SP (+)	293	0.116	0.0049	0.091	0.056	0.146					
	SP (-)	247	0.160	0.0066	0.138	0.091	0.197					
Latrunculin A 5-10 min	in SP area SP (+)	211	0.115	0.0053	0.091	0.059	0.152	p=0.094	p<0.001	p<0.001	p=0.616	p=0.071
	no SP area SP (+)	396	0.110	0.0043	0.089	0.056	0.135					
	SP (-)	439	0.140	0.0045	0.112	0.076	0.182					
Latrunculin A 15-20 min	in SP area SP (+)	92	0.129	0.0083	0.115	0.072	0.172	p=0.309	p=0.375	p<0.001	p<0.01	p<0.05
	no SP area SP (+)	262	0.133	0.0066	0.096	0.056	0.197					
	SP (-)	359	0.137	0.0054	0.104	0.058	0.189					
